# Experiences with long-term care for geriatric patients by an interprofessional outpatient care approach – a qualitative study

**DOI:** 10.1186/s12877-023-03809-1

**Published:** 2023-02-17

**Authors:** Denise Wilfling, Jona Budke, Nicole Warkentin, Katja Goetz

**Affiliations:** Institute of Family Medicine, University Hospital Schleswig-Holstein, Campus Luebeck, Ratzeburger Allee 160, 23538 Luebeck, Germany

**Keywords:** Long-term care, Care and case management, Geriatric patients, Interprofessional collaboration, Qualitative study

## Abstract

**Background:**

Outpatient care for geriatric patients is complex and requires the collaboration of different professions for supporting long-term care. Care and case management (CCM) could provide support with that. The long-term care of geriatric patients could be optimized with an interprofessional, cross-sectoral CCM. Therefore, the aim of the study was to evaluate the experiences and attitudes of those involved in the care with regard to the interprofessional design of the care for geriatric patients.

**Methods:**

A qualitative study design was used. Focus group interviews were conducted with those involved in the care (general practitioners (GP), health care assistants (HCA) as well as care and case managers (CM)). The interviews were digitally recorded, transcribed and analysed by qualitative content analysis.

**Results:**

Overall, ten focus groups were conducted in the five practice networks with *n* = 46 participants (*n* = 15 GP, *n* = 14 HCA and *n* = 17 CM). The participants evaluated the care they received from a CCM positively. The HCA and the GP were the primary points of contact for the CM. The close collaboration with the CM was experienced to be rewarding and relieving. Through their home-visitations, the CM gained a deep insight into the homelives of their patients and were thus able to accurately reflect the gaps in the care back to the family physicians.

**Conclusions:**

The different health care professionals involved in this type of care experience that an interprofessional and cross-sectoral CCM is able to optimally support the long-term care of geriatric patients. The different occupational groups involved in the care benefit from this type of care arrangement as well.

**Supplementary Information:**

The online version contains supplementary material available at 10.1186/s12877-023-03809-1.

## Background

Since the population is aging, the proportion of geriatric patients within the healthcare system is increasing as well [1]. The multimorbidity within this patient group necessitates a complex need for care [2]. The professions involved in the care are strongly challenged by this complexity [2]. General practitioners (GP) in particular are confronted by these challenges, which are amplified by the fact, that elderly patients prefer to stay within the confines of their own homes for as long as possible [2, 3]. In order to meet the necessary demands for care and to avoid the hospitalisation, rehospitalisation and institutionalisation of geriatric patients for as much as possible [4], it is recommended to take a coordinated and integrated approach to care based on the collaboration of different professional groups [5, 6].

Care and case management (CCM) could be an important component in that process [6]. In order to address the individual requirements of the patients [7], it is recommended to provide different interventions for the assessment, planning, coordination, monitoring and evaluation of the care. It has been demonstrated that CCM could help to meet the complex care requirements of geriatric patients [8–10].

The project RubiN (Regional ununterbrochen betreut im Netz; Continuous care in a regional network) is a CCM intervention, which follows this idea and is designed for geriatric patients over 70 years of age and their relatives and caregivers. Five practice networks implemented CCM for their geriatric patients. The patients received CCM provided by a specially trained care and case manager (CM). The CM assessed, coordinated and monitored the established care of the geriatric patients in close consultation and collaboration with the GP, who is a part of the practice network together with the CM. The health care assistants (HCA) work in the GP’s practice and support the CM as well as the GP in coordinating the patient’s care. Detailed information about that project and the intervention itself is available at Gloystein et al. [11]. Even though integrated and interprofessional care is recommended for geriatric patients, little is known about the experiences with this kind of collaboration, the related challenges and attitudes of those involved [12]. By definition, interprofessionalism is defined as the collaboration of different professions, even across professional borders if necessary, according to patient needs [13]. As stated in the literature, a precondition of successful collaboration is teamwork, which is based on respect and trust [14].

Therefore, the aim of this qualitative study was to evaluate the experiences and attitudes about the collaboration of the different professional groups involved in the long-term care for geriatric patients which was a part of the evaluation of the project RubiN [11].

## Methods

### Design

A qualitative study design was used. The reporting guideline COREQ (COnsolidated criteria for REporting Qualitative research) served as the foundation for the drafting of the manuscript [15] (please see additional file 1). Qualitative research is particularly well-suited to identify subjective viewpoints and experiences from the perspectives of the participants [16]. Focus group interviews were conducted with the participants of RubiN who were involved in the care (GP, HCA and CM). In doing so, the participants could mutually benefit and complement each other in the context of the group with their statements [17].

### Recruitment

The participants in the focus groups were people from the practice networks who participated in RubiN. The focus group participants were recruited from June to July 2020 by the project coordinators of their respective practice networks. Overall, 48 people consented in participation of the focus groups. Due to the fact that an interprofessional team approach was a key element in the care of the geriatric patients the composition of the participants in the groups should be as heterogenous as possible and consisted of GP, HCA and CM, who all worked in the practice network. In order to get a deeper insight on how the care for the geriatric patients was performed within the project RubiN, it was necessary to gain the different perspectives of each health care professional within the evaluation. Informed consent was obtained by a signed form in advance of participation. Each participant received a financial incentive of 50 Euros.

### Data collection and analysis

A semi-structured interview guide was developed by an interdisciplinary team consisting of a physician (NW) and a health care researcher (KG) in consultation with representatives from the practice networks. Both were female. The guideline contains questions about their experiences with regard to the exchange of information and collaboration within the bounds of interprofessional care for geriatric patients in RubiN, as well as the perceived changes which developed through such a care. The interview guide developed for this study is provided as Additional file 2. The guide was piloted with the first focus group and afterwards some individual formulations as well as the order of questions were slightly modified. Since the results of the first focus group did not differ significantly from the others, they were also included in the evaluation.

The focus groups were conducted face-to-face between August and September 2020 within the facilities of their respective practice networks. The focus groups were carried out in the five practice networks, which were all located in different regions (western-, northern- and eastern) in Germany. An equal distribution of the number of focus groups per region was desired as well as a sufficient number of participants per group, who were all responsible for the geriatric care within the five practice networks. Therefore, ten focus groups were formed with the group sizes varying between three to seven participants, two focus groups per practice network. The audio of the group discussions was recorded digitally, transcribed fully and anonymized. The analysis was done according to the qualitative content analysis of Mayring [18] using the analysis software Atlas.ti and a deductive- inductive approach was utilized. Following the guide, a temporary (deductive) category framework was created and over the course of the evaluation, it was either adjusted based on the content of the transcripts or new categories were added (inductive). Initially, the individual researchers (JB, DW) coded the transcripts independently from each other. The results were compared and discussed in regular consensus meetings (JB, DW, KG). By doing this as well as a detailed documentation of the research process, the criterion of intersubjective traceability, and with it a significant quality criterion for qualitative research, was fulfilled [19].

## Results

### Sample Characteristics

Table 1 is showing the socio-demographic data of the participants. Overall, ten focus groups were formed in the practice networks with *n* = 46 participants (*n* = 15 GP, *n* = 14 HCA, *n* = 17 CM). Two GPs cancelled their participation due to a lack of time. The majority of the CM were trained nurses. On average, the focus group discussions lasted around 75.6 min (± 13.5).

**Table 1:** Characteristics of the study participants (*n* = 46)

Characteristics	Details
Age in years, mean (SD)	46.6 (20.5)
Gender, female, *n* (%)	37 (80.4)
Function in the medical practice network, *n*	
General practitioner	15
Health care assistant	14
Care and case manager	17
Years of working in the medical practice network n*	
Less than 1 year	1
1 to 5 years	13
6 to 10 years	8
More than 10 years	7

^*^varies due to missing values, SD standard deviation

### Key categories

The experience and attitudes regarding the collaboration and exchange of information between the participants as well as the perceived changes related to this type of care and collaboration were discussed in the focus groups. Important aspects were illustrated with examples of the participants (GP, HCA, CM) from the focus groups (FG). Both code groups as well as the corresponding codes were illustrated in Table 2.

**Table 2:** Experiences and attitudes regarding interprofessional collaboration: main categories and sub-categories

Main category	Sub-categories
Collaboration and exchange of information	Evaluation of the interprofessional collaboration
	Exchange of information
	Collaboration within the practice network
	Collaboration outside of the practice network
Perceived changes to the care	The burden of the general practitioners
	The burden of care services
	Support of the care at home
	Satisfaction of patients
	Feeling of security
	Increased sensitization of geriatric care

### Collaboration and exchange of information

#### Evaluation of the interprofessional collaboration

The interprofessional collaboration was described positively by nearly all participants. Due to the constant exchange of information between the health care providers at eye level and appreciative interaction with each other a strong foundation of trust was created in each team, which was seen as essential for a good, interprofessional collaboration and was judged to be valuable by the GP and CM.

“It’s also a type of trust, which is extended to us by the family physician, when I say: “That’s the situation at home and it’s – the medicine is not properly taken”. For example, and “This is something I would definitely recommend”. And then always, they always – that was okay then, “of course, we will do that.”” (RubiN_FG4_CM).

### Exchange of information

The exchange of information between the participants involved in the care was described as “friendly cooperation”. When there was a need for clarification or an acute adjustment and anomalies, that was discussed together. When specific changes in geriatric care of patients were necessary, face-to-face case conferences were held. GP were also in favour of this approach, as this meant a certain amount of relief for them. Sufficient time was set aside for case conferences. As such, important cases and open questions could be discussed within the interprofessional team.

“Well, this was a very important point, that these conversations about the patients were held without being pressed for time, if possible. And I have to say: that took a lot of time. That’s how it is. But that somehow felt like it was very valuable.” (RubiN_FG2_GP).

### Collaboration within the practice networks

GP value the work of CM a lot and they experience a certain amount of relief thanks to the excellent collaboration, as a lot of non-medical decisions were made without their involvement or because their involvement was often unnecessary.

“That went well. And I knew: They are taken care of, the patients. I have heard: “RubiN was there” or I have heard from the < HCA > , that they became active there. That means, it happened practically around me or rather not behind my back, but simply taking away my work. And now I had, I didn’t have to appear anywhere.” (RubiN_FG6_GP).

The collaboration within the practice networks was described by the CM as close as well, which led to a good and trustworthy exchange and built a strong relationship within the care team. They described themselves as an “extended arm” of the family physician of sorts, since they could take more time with the patients and as such enabled an optimal addition to a mostly purely medical-therapeutic viewpoint. The open communication with those involved in the care as well as the mutual appreciation and recognition were emphasized. As a result, the collaboration was mutually respectful. While there were clear responsibilities, at the same time, those responsibilities were closely intertwined.

### Collaboration outside of the practice networks

The collaboration outside of the practice networks was described as predominantly positive. The CM had the impression, that their work outside of the practice networks was greatly appreciated, even though it had seemed that at the beginning, some service suppliers had reservations.

“Well, they just know our work by now, that has been established somewhere. And, well, we are doing lots and lots of networking.” (RubiN_FG6_CM).

The networking outside the practice networks focused on active involvement on the community level such as sport groups for elderly, local nursing homes and ambulatory care in order to support the geriatric patients. Thanks to the long-time collaboration with the different service providers, the initial concerns and competitiveness could be alleviated. The expertise of the CM was appreciated and recognized. The CM described, that according to them, a great network of different service providers came to exist over time, who were frequently in contact with each other in order to provide the best possible care for their patients.

### Perceived changes to the care

#### The burden of the general practitioners

Most practices initially described the phase of recruitment of geriatric patients for this project as demanding. Over time, it became clear how much of a relief the services of the CM provided to the GP.

“It was a great relief, since we didn’t have to do things then…We didn’t have to come up with it ourselves, since we get something recommended. […] and they are practically doing it by themselves and are a great addition to the whole.” (RubiN_FG6_GP).

It was remarkable, that GP as well as HCA had the impression that ever since a CM had been established, many patients had significantly fewer visits to medical practices, even though this could not be explicitly and quantitatively measured. There were certain doubts about illnesses or questions towards the care which could be alleviated with regular contact with the CM. The networking that was done within the framework of the implementation of the care model was highlighted, since it not only made the jobs of the GP significantly easier, it also somehow improved it for them, since they did not have to take care of every aspect of the care anymore. As such, the GP had the opportunity to primarily focus on the basic medical issues, since the coordination of care was handled by other actors within the care model.

### The burden of care services

The burden for outpatient care services was reduced as well, since a lot of activities, such as the conduction of diverse geriatric assessments, were done by the CM and this saved the care services a lot of time. The CM also felt that a lot of problems and questions the patients otherwise would have directed towards the care services were now being intercepted by the CM since the CM were now additionally available points of contact.

“And it was a relief for the care services as well, because they said: “It’s great, when you do it” and then they have less paperwork to do and they, of course, also have a patient who is satisfied.” (RubiN_FG2_CM).

### Support of the care at home

It was possible, through the insight into the homelife of the patients, to implement appropriate support options, which supported their ability to remain in their own homes. The support of the care at home included, among other things, adjustments to the living spaces to be more handicapped-accessible, the implementation of aid utilities, the inclusion of outpatient care services, the organization of support services to assist with shopping or to order meals on wheels.

### Satisfaction of patients

Most of the participants perceived the satisfaction of the patients with the care model. The interprofessional design of the care offered patients the option to contact someone, in addition to their family physician, to talk about their own health and problems. The participants stated that they perceived a positive impact on the well-being of the patients.

“Well, the conversation, to also talk about things which maybe we would never have gotten around to. Suddenly, they are seeing the light of day. And that is also a form of well-being for the patients. Principally speaking, we do want self-determination and stability.” (RubiN_FG2_CM).

Patients consistently gave positive feedback to the CM and they were all grateful to have participated in the project and that there was an additional qualified individual at their side alongside their family physician. The care model alleviated their fears and reduced their burdens, as they gained the impression, that whenever it became necessary, someone extended a helping hand. At the same time, as was described previously, the care allowed for their self-determination to be protected and supported. The interprofessional care model made it possible to support the patients when and where they required assistance.

### Feeling of security

Most of the people involved in the care could feel a sense of security. GP as well as CM reported, that the patients gained a stronger sense of security thanks to the regular contact with the CM, since they had a fixed person of contact who was available at all times.

“It’s reassuring after all, if someone is approached and then they can pour out their heart, so to say, well to find someone very trustworthy. That is a sense of security as well, which you gain when you know: you can always call her, she listens to me, she will come over, I can talk with her about it.” (RubiN_FG6_GP).

GP realized that the patients felt more secure when someone came over to their homes regularly, who worked closely with their family physician, which, in turn, also created a sense of security within the GP. Even the CM described a sense of security, which resulted from the fact that they were following a clearly defined work- and care contract, where they experienced optimal support through the excellent collaboration with an interprofessional team.

### Increased sensitization of geriatric care

GP noted, that by participating in the project, they increased their sensitization about the topic of geriatric care and that they gained a certain overview on the subject. That includes the prescription of medicine and aid utilities or applying for financial help such as care degree or the application for various permits in a timely manner.

“Because I believe, I am a lot more sensitized now, even now, when the project is over, I am still considering early on: What else do I have to consider, outside of medicine and physiotherapy and something like that?” (Rubin_FG2_GP).

## Discussion

The aim of this qualitative study was to evaluate the experiences and attitudes of those involved in the long-term care for geriatric patients with regard to an interprofessional design. The literature shows, that well-functioning collaboration is an essential prerequisite for optimal care [20]. The results of this study are in line with international results. The collaboration was regarded as excellent from outside the practice networks as well as from within. It can be assumed that the participation in the care model could be seen as valuable for the long-term care of these patients.

The mutual trust between the individual professions was named to be basis of this well-functioning collaboration. For this basis of trust to exist, everyone involved had to be aware of their own responsibilities as well as the roles of everyone else and they had to acknowledge and value the competency and accomplishments of others. Gregory et al. [21] and Pullon [22] also reached this conclusion, that the mutual respect combined with mutual trust was identified to be the core element of interprofessional collaboration.

Furthermore, the different health professionals experience that through the interprofessional care they offered, patients could be supported in their long-term care especially to live at home as long as possible. This holistic approach includes the insight into the individual living situations and the living environment of the patients. By doing so, preventative measures could be taken earlier and it was possible for the patients to remain in their own homes and avoid in-patient stays or necessary institutionalization. Golden et al. [4] emphasized this value of the integrated care. The cooperation of the various professional groups and thus the interprofessional approach made it possible to take the physical, psychological and social requirements of geriatric patients into consideration, as different responsibilities and competencies meet and complement each other, due to the different professions involved in the care. As such, physical problems were handled by family physicians, while psychological and social needs were mostly transferred to the responsibilities of the CM.

This in turn had a positive effect on patient satisfaction as well as their quality of life [23]. Family physicians in particular could perceive changes thanks to the interprofessional design of the care. For one, it led to a reduction of their workload, since patients tended to visit GP less. For the other, they became more sensitized about the topic of geriatric care. This can be seen in situations, where some geriatric patients might have a higher necessity for care than can be initially apparent in the practice of the family physician. The support of the CM helps to broaden the perception of family physicians and at the same time strengthens the interprofessional collaboration and makes it possible to optimize the care for geriatric patients in the outpatient sector [24].

Moreover, the participants in our study reported that patients gained a stronger believe of security, which is in the line with the perspective of patients on the project RubiN as an interview study previously showed. The structural and functional support that geriatric patients received by a CM lead to a sense of security [25]. It can be assumed that beside a GP a CM who could be responsible for coordination of care by such patient groups could help in reducing the burden of care. Therefore, more research is needed to evaluate the benefit of the implementation of a CM in the care process.

### Limitations of the study

To answer the research question a qualitative study design was chosen. The participants in this study were directly involved in project RubiN and were recruited by the project coordinators of the different practice networks. The number of participants (GPs, HCAs and CMs) who were contacted by the project coordinators before starting the focus group was unknown. A selection bias as well as a social response behaviour cannot be excluded due to that the participants were very positive in their statements. The statements within focus groups could include power issues related to hierarchy due to the heterogeneous composition of the groups. However, the moderator of the focus groups encouraged to feel safe and to be open in their statements. Next to the reimbursement, it is also possible that only people who were motivated or satisfied with project RubiN participated in the focus groups.

## Conclusions

The focus group study shows that the GPs, HCA and CM experience this kind of collaboration as positive for the long-term care for geriatric patients. The different health professionals demonstrate that the positive attitude considering interprofessional collaboration can help in gaining security for patients and trust. Furthermore, the results of this study emphasize the importance of well-functioning and interprofessional collaboration for an optimized quality of care especially for long-term care of geriatric patients. Open and appreciative communication and collaboration between those involved in the care is required in order to create the necessary level of trust, which creates a sense of security in those involved in the care and which is ultimately mirrored by the patients. This thesis provides references that an integrated care model can bridge sectoral borders and interfaces if the different professions and service providers collaborate well with each other. At the same time, integrated care can reduce the burden on family physicians, which is perceived as very high since geriatric patients require a complex level of care. It can be assumed that other patient groups with a chronic condition could have a benefit from this type of care in order to ensure an adequate quality of care. Moreover, the implementation of a CM for the coordination of care for specific patient groups could reduce the burden of care for GPs.

## Supplementary Information

**Additional file 1.** Completed form of the COREQ-guideline.

**Additional file 2. **Main questions of the focus group discussions.

## Data Availability

The transcripts of the focus group interviews used are available from the corresponding author on reasonable request.

## Abbreviations

CM: Care and case manager
CCM: Care and case management
FG: Focus groups
GP: General practitioners
HCA: Health care assistants
SD: Standard deviation
RubiN: Regional ununterbrochen betreut im Netz; Continuous care in a regional network
