# Supplementary material for: Experiences with long-term care for geriatric patients by an interprofessional outpatient care approach – a qualitative study
Source: BMC Geriatr. 2023 Feb 17;23:102. doi: 10.1186/s12877-023-03809-1 (PMC9938618; doi:10.1186/s12877-023-03809-1)
Supplement: Supplementary file 1 — Additional file 1. Completed form of the COREQ-guideline. [file 12877_2023_3809_MOESM1_ESM.pdf]

Table 1 Consolidated criteria for reporting qualitative studies (COREQ): 32-item checklist

No	Item	Guide questions/description
**Domain 1: Research team and reflexivity**		
Personal Characteristics		
1.	Interviewer/facilitator	Which author/s conducted the interview or focus group? Methods, p5, paragraph 1
2.	Credentials	What were the researcher's credentials? *E.g. PhD, MD* Methods, p5, paragraph 1
3.	Occupation	What was their occupation at the time of the study? Title page
4.	Gender	Was the researcher male or female? Methods, p5, paragraph 1
5.	Experience and training	What experience or training did the researcher have? Methods, p5, paragraph 1
Relationship with participants		
6.	Relationship established	Was a relationship established prior to study commencement? N/A
7.	Participant knowledge of the interviewer	What did the participants know about the researcher? e.g. *personal goals, reasons for doing the research* Background, p.3 – 4, last paragraph & paragraph 1
8.	Interviewer characteristics	What characteristics were reported about the interviewer/facilitator? e.g. *Bias, assumptions, reasons and interests in the research topic* Limitations of the study, paragraph 1 p. 14
**Domain 2: study design**		
Theoretical framework		
9.	Methodological orientation and Theory	What methodological orientation was stated to underpin the study? *e.g. grounded theory, discourse analysis, ethnography, phenomenology, content analysis* Methods, p5, paragraph 2
Participant selection		
10.	Sampling	How were participants selected? *e.g. purposive, convenience, consecutive, snowball* Methods, p4, parag 3
11.	Method of approach	How were participants approached? e.g. *face-to-face, telephone, mail, email* Methods, p4, paragr. 3
12.	Sample size	How many participants were in the study? Results, p6, paragraph 1
13.	Non-participation	How many people refused to participate or dropped out? Reasons? N/A
Setting		
14.	Setting of data collection	Where was the data collected? e.g. *home, clinic, workplace* Methods, p5, paragraph 2
15.	Presence of non-participants	Was anyone else present besides the participants and researchers? No
16.	Description of sample	What are the important characteristics of the sample? *e.g. demographic data, date* Results, p6, paragraph 1, Table 1
Data collection		
17.	Interview guide	Were questions, prompts, guides provided by the authors? Was it pilot tested? Methods, p.5, par. 1
18.	Repeat interviews	Were repeat interviews carried out? If yes, how many? Methods, p.5, paragraph 2
19.	Audio/visual recording	Did the research use audio or visual recording to collect the data? Methods, p.5, paragraph 2
20.	Field notes	Were field notes made during and/or after the interview or focus group? N/A
21.	Duration	What was the duration of the interviews or focus group? Results, p.6, paragraph 1
22.	Data saturation	Was data saturation discussed? Methods, p.5, last paragraph
23.	Transcripts returned	Were transcripts returned to participants for comment and/or correction? N/A
**Domain 3: analysis and findingsz**		
Data analysis		
24.	Number of data coders	How many data coders coded the data? Methods, p.5, last paragraph
25.	Description of the coding tree	Did authors provide a description of the coding tree? Results, p.6, paragraph 2
26.	Derivation of themes	Were themes identified in advance or derived from the data? Results, p.6, paragraph 2
27.	Software	What software, if applicable, was used to manage the data? Methods, p.5, last paragraph
28.	Participant checking	Did participants provide feedback on the findings? N/A
Reporting		
29.	Quotations presented	Were participant quotations presented to illustrate the themes / findings? Was each quotation identified? e.g. *participant number* Results, p.6 – 12
30.	Data and findings consistent	Was there consistency between the data presented and the findings? yes
31.	Clarity of major themes	Were major themes clearly presented in the findings? Results, p.6 – 12
32.	Clarity of minor themes	Is there a description of diverse cases or discussion of minor themes? N/A
