# Supplementary material for: Experiences with long-term care for geriatric patients by an interprofessional outpatient care approach – a qualitative study
Source: BMC Geriatr. 2023 Feb 17;23:102. doi: 10.1186/s12877-023-03809-1 (PMC9938618; doi:10.1186/s12877-023-03809-1)
Supplement: Supplementary file 2 — Additional file 2. Main questions of the focus group discussions. [file 12877_2023_3809_MOESM2_ESM.pdf]

Interview guideline for the focus group discussion

- Main questions -

How would you assess the collaboration among each other?

How would you assess the collaboration with the care and case managers?

How did the exchange of information between the different health care providers work out?

To what changes led your participation on the project RubiN for the health care of geriatric patients?

How would you assess these changes?
